# Genetic polymorphism among Cryptosporidium parvum isolates: evidence of two distinct human transmission cycles.

**DOI:** 10.3201/eid0304.970423

**Published:** 1997

**Authors:** M M Peng, L Xiao, A R Freeman, M J Arrowood, A A Escalante, A C Weltman, C S Ong, W R Mac Kenzie, A A Lal, C B Beard

**Affiliations:** University of Michigan, Ann Arbor, USA.

## Abstract

We report the results of molecular analysis of 39 isolates of Cryptosporidium parvum from human and bovine sources in nine human outbreaks and from bovine sources from a wide geographic distribution. All 39 isolates could be divided into either of two genotypes, on the basis of genetic polymorphism observed at the thrombospondin-related adhesion protein (TRAP-C2) locus. Genotype 1 was observed only in isolates from humans. Genotype 2, however, was seen in calf isolates and in isolates from a subset of human patients who reported direct exposure to infected cattle or consumed items thought to be contaminated with cattle faces. Furthermore, experimental infection studies showed that genotype 2 isolates were infective to mice or calves under routine laboratory conditions, whereas genotype 1 isolates were not. These results support the occurrence of two distinct transmission cycles of C. parvum in humans.


### Genetic Polymorphism Among *Cryptosporidium parvum* Isolates: Evidence of Two Distinct Human Transmission Cycles

We report the results of molecular analysis of 39 isolates of *Cryptosporidium parvum* from human and bovine sources in nine human outbreaks and from bovine sources from a wide geographic distribution. All 39 isolates could be divided into either of two genotypes, on the basis of genetic polymorphism observed at the thrombospondin-related adhesion protein (TRAP-C2) locus. Genotype 1 was observed only in isolates from humans. Genotype 2, however, was seen in calf isolates and in isolates from a subset of human patients who reported direct exposure to infected cattle or consumed items thought to be contaminated with cattle feces. Furthermore, experimental infection studies showed that genotype 2 isolates were infective to mice or calves under routine laboratory conditions, whereas genotype 1 isolates were not. These results support the occurrence of two distinct transmission cycles of *C. parvum* in humans.

Long considered a veterinary disease, cryptosporidiosis has emerged as an important infectious disease in humans (1). In immunocompetent persons, the disease is usually self-limiting; however, in the immunocompromised, it is frequently chronic, more severe, and sometimes fatal. Cryptosporidiosis is one of the major secondary diagnoses in people with AIDS and is associated with a twofold greater hazard of death than other AIDS-defining diagnoses (2).

A number of major waterborne outbreaks of cryptosporidiosis have occurred in urban settings (3); however, the disease also occurs sporadically. Since most *Cryptosporidium parvum* infections are self-limiting and symptomatically similar to other diarrheal diseases, the disease may often be undiagnosed or misdiagnosed in the absence of a recognized outbreak. Consequently, the actual incidence of cryptosporidiosis and the relative importance of each of its many modes of transmission are largely unknown. For these reasons, laboratory tools are needed for quantitative and qualitative environmental sampling and for strain analysis of *Cryptosporidium* isolates. These tools would be extremely valuable for source identification and outbreak investigations, for correlation with clinically important phenotypes, and for determining risk factors in nonepidemic settings.

A number of new nucleic acid-based approaches have been developed for detection, diagnosis, and typing of *C. parvum*, among them polymerase chain reaction (PCR)-based tests that focus either on random amplification of DNA polymorphisms or on specific polymorphic genetic loci (4-7). These PCR-based tests suggest the existence of strain variation and the possibility of two distinct transmission cycles among *C. parvum* isolates that infect humans. In this study, we examined genetic polymorphism among *C. parvum* isolates from human and nonhuman sources to identify strain-specific markers that could be correlated with epidemiologically important phenotypes.

### Analytic Approach

### Parasite Isolates

Thirty-nine isolates were examined from stool samples positive for *C. parvum:* 17 were obtained from humans or calves during outbreaks in the United States and Canada (Table 1); one was a calf isolate (Iowa calf) routinely passaged in neonatal Holstein calves in our laboratories; and 21 were obtained from cattle from Georgia, Alabama, Ohio, Oklahoma, Kansas, Iowa, Idaho, Utah, and Washington. All samples were collected and placed directly into a

Table 1. *Cryptosporidium parvum* isolates examined

| Isolate | Implicated source | Host | Ref. |
|---|---|---|---|
| Maine,1993 | apple cider | human | 16 |
| Wisconsin, 1993 | drinking water | human | 3 |
| Wisconsin, 1996 | drinking water | human | * |
| Georgia (day care), 1995 | person-to-person | human | * |
| Georgia (water-park), 1995 | recreational water | human | 17 |
| Florida, 1995 | drinking water | human | 18 |
| British Columbia, 1996 | drinking water | human | 15 |
| Texas, 1996 | unknown | human | * |
| Pennsylvania, 1997 | bovine contact | human, calf | * |

*Reference is this article.

2.5% potassium dichromate solution and were stored at 4°C. Oocysts were purified by using discontinuous sucrose and Percoll or cesium chloride gradients (8,9).

### Isolation of Genomic DNA

Parasite DNA was isolated as described by Kim et al. (10). Briefly, oocysts were ruptured by using five freeze-thaw cycles (dry ice ethanol bath and 65°C) in a lysis buffer (120 mM NaCl, 10 mM EDTA, 25 mM Tris pH 7.5, 1% Sarkosyl) containing proteinase K. The samples were incubated for 1 hour at 55°C to inactivate nucleases. Then DNA was extracted with phenol/chloroform/isoamyl alcohol (25:24:1) and chloroform/isoamyl alcohol (24:1), precipitated with absolute ethanol, washed with 70% ethanol, and resuspended in TE buffer (10 mM Tris pH 8.0, 1 mM EDTA).

### PCR Amplification and Sequencing and Analysis

The gene fragment of interest, a 369-bp region of the thrombospondin-related adhesive protein (TRAP-C2) of *C. parvum*, was amplified with the following primers: 5'-CAT ATT CCC TGT CCC TTG AGT TGT-3' and 5'-TGG ACA ACC CAA ATG CAG AC-3', which correspond, respectively, to positions 812 to 835 on the coding strand and positions 1,161 to 1,180 on the negative strand, of GenBank sequence X77586. The reactions were performed with Perkin-Elmer (Perkin-Elmer Corporation, Foster City, CA) PCR reagents, including 1X PCR buffer, 2.5 mM $MgCl_2$, 0.2 mM each dNTP, 0.4 mM of each specific primer, and 2.5 U of Taq DNA polymerase. After a 1-minute hot start at 94°C, the reactions went through 35 to 40 cycles of denaturing at 94°C for 30 seconds, annealing at 45°C for 30 seconds, and extension at 72°C for 1 minute, followed by a 72°C incubation for strand completion.

An aliquot of each PCR product was examined by agarose gel electrophoresis; the remaining PCR product was purified by using the Wizard PCR Prep Kit (Promega Corporation, Madison, WI). Purified PCR fragments were sequenced directly on an ABI 377 automated sequencer by fluorescent cycle sequencing using dye-terminator chemistry with AmpliTaq FS (Perkin-Elmer-Applied Biosystems) according to the manufacturer's recommended procedure. The same primer sets used initially for PCR were used again for sequencing, diluted to a concentration of 10 pMoles in the final sequencing reaction. Downstream analysis of sequence data was accomplished by using the Sequence Navigator program (Perkin–Elmer-Applied Biosystems). Multiple sequence alignments were performed by using the Pileup program (11). Animal isolates were manually sequenced by the dideoxy chain-termination method (12), using the Sequenase Version 2.0 kit (J.T. Baker, Phillipsburg, NJ) with the sequences of a few isolates confirmed by automated sequencing.

### Experimental Infection Studies

Purified oocysts ranged in age from 1 to 6 months at the time of inoculation of cell cultures or animals. This age range is well within the storage time that maintains oocyst viability and infectivity (e.g., laboratory-passaged isolates are 40% to 50% viable after storage for 6 months). Approximately $10^6$ oocysts were administered orally to 2-day-old calves or to 4- to 6-day-old BALB/c or SCID mice by using established procedures (8,13). Beginning at day 5, stools were collected and examined daily by light microscopy or by immunofluorescent flow cytometry for *C. parvum* oocysts (14).

### Findings

### Sequence Determination and Analysis

A single specific band of 369 bp, corresponding to bases 812 to 1180 of the 1.1 kb *C. parvum* TRAP-C2 gene (GenBank accession number

X77586) was amplified from 39 different isolates (Figure 1). Although the sequence similarity was very high among all gene fragments, multiple alignments showed two primary genotypes. These genotypes could be established on the basis of nucleotide substitutions at five independent positions, three being silent changes and the other two resulting in amino acid changes (Figure 2). Of the five changes, four were transitions, and one was a transversion.

Genotype 1 included human isolates from Wisconsin, Georgia, Florida, and Texas. Genotype 2 contained human isolates from Maine and British Columbia, human and calf isolates from Pennsylvania, the Iowa calf laboratory strain, and 21 bovine isolates from various locations around the country. Two human isolates (one from Florida and the Texas isolate) appeared to represent a variant of genotype 1. In both cases, they shared the first four positions with the other genotype 1 isolates. The fifth position, however, was the same as that of genotype 2 isolates.

### Experimental Infection Studies

The results of experimental infection studies are shown in Table 2. The genotype 2 isolates from human outbreaks in Maine and Pennsylvania and from a calf in Iowa all readily infect both mice and calves. The genotype 2 isolate from British Columbia was also reported to be infective to immunosuppressed C57BL/6 mice (15). None of the genotype 1 isolates from humans—from Wisconsin, Florida, a Georgia day-care facility, and a Georgia water park—could be established in either mouse or calf. A single sample, the Georgia day-care isolate, was examined for its ability to infect a neonatal pig. This isolate caused a brief moderate infection in a neonatal pig host (data not shown) but not in calves or mice. One of the Wisconsin isolates and the Georgia day-care isolate were tested for their infectivity to MDCK cell cultures; both successfully infected this cell line (data not shown).

| | Position: | 15 | 42 | 64 | 111 | 244 |
|---|---|---|---|---|---|---|
| | Isolate: | | | | | |
| | **Genotype 1** | | | | | |
| Human Isolates | Milwaukee 93 /1 | G | C | T | C | T |
| | Milwaukee 93 /2 | G | C | T | C | T |
| | Milwaukee 93 /3 | G | C | T | C | T |
| | Milwaukee 96 | G | C | T | C | T |
| | Georgia-DC 95 | G | C | T | C | T |
| | Georgia-WP 95 /1 | G | C | T | C | T |
| | Georgia-WP 95 /2 | G | C | T | C | T |
| | Florida 95 /1 | G | C | T | C | T |
| | Florida 95 /2 | G | C | T | C | T |
| | Florida 95 /3 | G | C | T | C | T |
| | Florida 95 /4 | G | C | T | C | T |
| | Florida 95 /5 | G | C | T | C | C |
| | Texas 96 | G | C | T | C | C |
| | **Genotype 2** | | | | | |
| | Maine Cider 93 | A | T | G | T | C |
| | B.C. Canada 96 | A | T | G | T | C |
| | Pennsylvania 97 (H) | A | T | G | T | C |
| Calf Isolates | Pennsylvania 97 (C) | A | T | G | T | C |
| | Iowa calf (CDC) | A | T | G | T | C |
| | Published calf | A | T | G | T | C |
| | Other bovine (n=21) | A | T | G | T | C |

Figure 1. Alignment of TRAP-C2 nucleotide positions that show polymorphism among *Cryptosporidium parvum* isolates from human and nonhuman sources. Published calf sequence refers to Genbank accession number X77586. Other bovine (n=21) refers to 21 samples (from Georgia, Alabama, Ohio, Oklahoma, Kansas, Iowa, Idaho, Utah, and Washington) that had the same genotype.

### Conclusions

All isolates examined in this study could be grouped easily into two distinct genotypes defined by nucleotide substitutions at five positions within the TRAP-C2 locus, with genotype 1 containing a variant at the fifth position that was represented by two isolates. All isolates in genotype 1 were from human stool. The isolates in genotype 2, however, were from both human and bovine sources. In the limited number of isolates that were tested in experimental infection studies, all genotype 2 isolates could be established readily in mice and calves. None of the genotype 1 isolates, however, could be shown to be infective to either of these hosts. The genotype and experimental infection data suggest the possibility of two distinct populations of *C. parvum* cycling in humans. One population appears to involve zoonotic transmission from calf-to-human with subsequent human-to-human and human-to-calf transmission. The other population appears to involve an anthroponotic transmission cycle, exclusively in humans. This hypothesis is consistent with the data from the epidemiologic investigations from which the isolates were obtained.

Genotype 2 characteristics were identified in human isolates from the Maine 1993, British Columbia 1996, and Pennsylvania 1997 outbreaks, and in all isolates from bovine sources. Both the Maine and Pennsylvania outbreaks could be directly linked to a calf source of *C. parvum*. The Maine outbreak was associated with contaminated apple cider (16). Interestingly, *C. parvum* oocysts were isolated directly from apple cider, the press used for preparing the

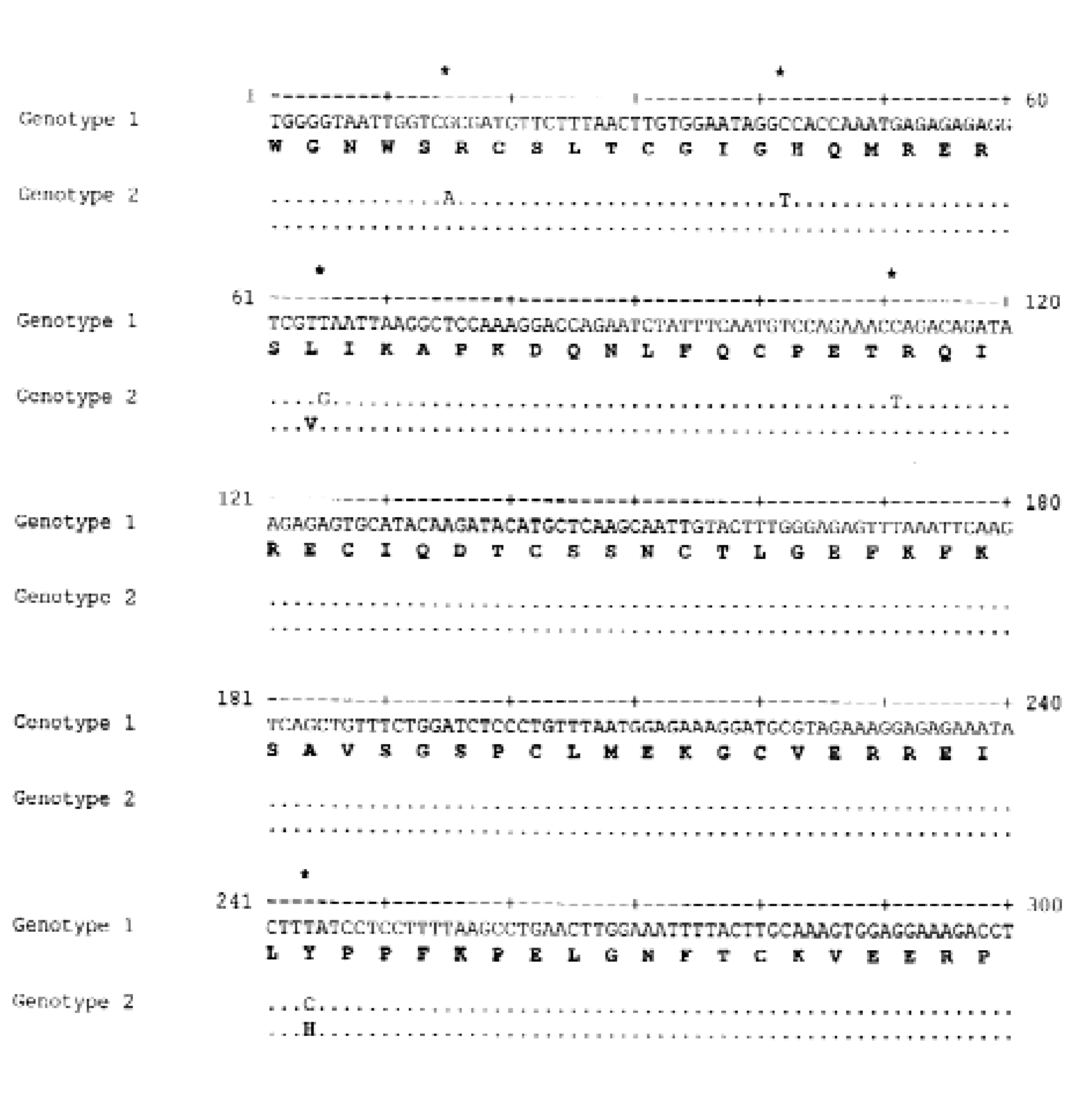

Figure 2. DNA and putative amino acid sequences of *Cryptosporidium parvum* TRAP-C2 genotypes 1 and 2.

cider, and a calf stool specimen from the farm that supplied the apples.

The Pennsylvania focus involved three families that together purchased three young calves that subsequently developed scours. Nine members of three families had diarrhea, and two were hospitalized. *C. parvum* oocysts were isolated from two calves and five humans; all isolates examined demonstrated the genotype 2 pattern.

The British Columbia isolate came from a human patient infected in an outbreak (approximately 2,000 cases) that occurred in the small rural community of Cranbrook (15). During the outbreak investigation, *Cryptosporidium* oocysts were identified in human fecal specimens, in cattle manure specimens found near the watershed, and in water samples from the reservoir intake.

Table 2. Experimental infection studies with *Cryptosporidium parvum* isolates from various sources

| Isolate | Experimental host | Infection |
|---|---|---|
| Maine, 1993 | mouse | + |
| | calf | + |
| Wisconsin, 1993 | mouse | - |
| | calf | - |
| Georgia (day care), 1995 | mouse | - |
| | calf | ND |
| Georgia (water park), 1995 | mouse | - |
| | calf | - |
| Florida, 1995 | mouse | - |
| | calf | ND |
| Iowa (bovine), 1984 | mouse | + |
| | calf | + |

ND = not done

Of the genotype 1 isolates examined, epidemiologic investigations were conducted for the Georgia water park 1995, Florida 1995, and Wisconsin 1993 outbreaks. In the Georgia water park outbreak, approximately 2,900 persons met the case definition for clinical cryptosporidiosis. In a sample of these patients, the following risk factors were evaluated in telephone interviews: swimming in lakes or pools, exposure to day care or to persons with diarrhea, contact with young animals, drinking water from various sources, chronic illness, and water park attendance. The only factor independently associated with diarrheal disease was water park attendance (17).

The Florida 1995 outbreak occurred at a day camp in central Florida and had approximately 70 cases (18). Risk factors examined included participating in camp activities, eating lunches provided at the camp, and drinking water from various specified sources. *C. parvum* oocysts were observed in the stools of 16 persons and in water from an outside tap. Fecal contamination (of unknown origin) of the tap was the suspected source of the outbreak. Five specimens were examined from this outbreak, all of which belonged to the genotype 1 grouping; one displayed additional polymorphism at nucleotide position 244 (Figure 1). The Texas 1995 isolate showed this same polymorphism, which we think is most accurately described as a subset of genotype 1.

The Wisconsin 1993 outbreak, which affected more than 403,000 people, is the largest waterborne disease outbreak ever recorded in the United States. Four isolates were examined, three isolated during the original outbreak and a fourth isolated in 1996 from an AIDS patient with a chronic infection who had initially been infected in the 1993 outbreak.

During the Wisconsin outbreak, possible sources of contamination of Lake Michigan with *Cryptosporidium* oocysts included cattle along two rivers that fed Milwaukee Harbor, slaughterhouses, and human feces (3,19). The genotypic and experimental infection data from the four isolates we examined suggest a human rather than bovine source. However, these results come from the analysis of only four samples from a massive outbreak, and the degree to which these samples are representative of the entire outbreak remains uncertain.

All genotype 2 isolates examined in this study came from persons that had direct links or potential exposure to *C. parvum* from an infected animal. All samples tested in experimental infection studies were also infective to both mice and calves. In the genotype 1 isolates, however, while the initial source of the cases was never directly determined experimentally, no confirmed links to bovine sources were found, but exposure to water contaminated with human feces could have occurred. Furthermore, of the isolates tested in experimental infection studies, none could successfully infect laboratory animals. These results lead us to suggest the possibility of a second transmission cycle that is anthroponotic and maintained through person-to-person contact or through human sewage contamination of the water supply.

The observations reported here with respect to genotypic variation among *C. parvum* isolates from humans and animals are very similar to those reported by other groups. These studies generally reported one allozyme pattern or genotype associated with human isolates and a second genotype or allozyme in bovine samples and a subset of human samples. The specific genes or regions examined differed in each study but included electromorphs of phosphoglucomutase and hexokinase (20), random amplified polymorphic DNA (RAPD) analysis of an unspecified region (4), a repetitive DNA sequence (6), the 18S rRNA gene and adjacent internal transcribed spacer (ITS) region I (5), the *Cryptosporidium* oocyst wall protein (COWP) locus (7), and the dihydrofolate reductase-thymidylate synthase (DHFR-TS) gene (21). At least two additional studies suggest the possibilities of two transmission cycles, on the basis of epidemiologic or experimental observations (22,23).

The TRAP-C2 protein is a member of a class of proteins present in all apicomplexans examined to date (24-26). This protein is associated with the cell surface and micronemal complex of these parasites and is thought to be involved in surface attachment; consequently, changes in this protein could affect attachment specificity and the resultant host range. Were this the case, such a mechanism could explain why the host range of one genotype might be different from that of a second genotype, resulting in distinct transmission cycles. In the two isolates that make up the variant of genotype 1, the T-to-C transition results in a change in the amino acid sequence from tyrosine to histidine. If variations in this protein affect host preference, the histidine-to-tyrosine change would have to be inconsequential with respect to protein function and host specificity. Observations in *Plasmodium* suggest that the WCSP motif in the TRAP gene is the functional domain involved in surface attachment; however, the polymorphism we observed in *C. parvum* did not involve this region. Additional studies are needed to clarify the relationship, if any, of polymorphism in this gene to host range.

The conclusion that two transmission cycles exist for *C. parvum* is now supported by the results of independent groups, using markers at six different genetic loci. This conclusion, if valid, may have important implications for the prevention and control of cryptosporidiosis in urban settings. Cattle have been the most commonly implicated source of water contamination in outbreaks outside the United States but not conclusively within the United States. Measures for preventing water contamination have in some cases included the removal of cattle from watershed areas in or around municipalities. If, however, sewer overflows and inadequate sewage treatment are the primary source of water contamination in urban settings where anthroponotic cycles are being maintained, focusing solely on cattle could fail to eliminate a very important source of infection.

The results of this study suggest the need to 1) combine the typing approaches of various groups into a multilocus approach for genetic typing of *C. parvum* that would result in a reliable and robust method for strain typing, 2) apply multilocus typing to a large number of *C. parvum* isolates both from epidemic and isolated cases and from a large geographic distribution to determine the prevalence of these two genotypes and their quantitative importance as indicators of specific risk factors, and 3) identify additional genetic loci that will allow more precise determination of strain variation and linkage of genotypic variation to specific clinical and epidemiologically important outcomes.

### Acknowledgments

We thank Thomas R. Navin, David G. Addiss, and Dennis D. Juranek, Centers for Disease Control and Prevention (CDC), for critical reading of the manuscript; Gisela Withers, Alfred L. Loban, Susan Yeager, Barbara Fisher, and Marshall P. Deasy, Pennsylvania Department of Health, Charles Brummit, St. Lukes Medical Center, Milwaukee, WI, and Michael J. Beach, CDC, for providing samples; Brian Holloway and staff, CDC, for providing DNA oligonucleotide primers; Robert W. Ryder, Yale University School of Medicine, and Mark L. Wilson, The University of Michigan, for help in coordinating the study; and Kimberley B. Donaldson, Donghyun Hahn, Kimberly Y. Won, Chunfu Yang, and Lillian Escalante, CDC, for technical support in completing the work.

**Michael M. Peng,* Lihua Xiao,† Amanda R. Freeman,† Michael J. Arrowood,† Ananias A. Escalante,† André C. Weltman,‡ Corinne S.L. Ong,¶ William R. Mac Kenzie,† Altaf A. Lal,† and Charles B. Beard†**

*The University of Michigan, Ann Arbor, Michigan, USA; †Centers for Disease Control and Prevention, Atlanta, Georgia, USA; ‡Pennsylvania Department of Health, Harrisburg, Pennsylvania, USA; and ¶University of British Columbia, British Columbia, Canada

### References

1. Guerrant RL. Cryptosporidiosis: an emerging, highly infectious threat. Emerg Infect Dis 1997;3:51-7.
2. Colford JM, Tager IB, Hirozawa AM, Lemp GF, Aragon T, Petersen C. Cryptosporidiosis among patients infected with human immunodeficiency virus. Am J Epidemiol 1996;144:807-16.
3. MacKenzie WR, Hoxie NJ, Proctor ME, Gradus MS, Blair KA, Peterson DE, et al. A massive outbreak in Milwaukee of *Cryptosporidium* infection transmitted through the public water supply. N Engl J Med 1994;331:161-7.
4. Morgan UM, Constantine CC, O'Donoghue P, Meloni BP, O'Brien PA, Thompson RCA. Molecular characterization of *Cryptosporidium* isolates from humans and other animals using random amplified polymorphic DNA analysis. Am J Trop Med Hyg 1995;52:559-64.
5. Carraway M, Tzipori S, Widmer G. Identification of genetic heterogeneity in the *Cryptosporidium*

*parvum* ribosomal repeat. Appl Environ Microbiol 1996;62:712-6.
6. Bonnin A, Fourmaux MN, Dubremetz JF, Nelson RG, Gobet P, Harly G, et al. Genotyping human and bovine isolates of *Cryptosporidium parvum* by polymerase chain reaction-restriction fragment length polymorphism analysis of a repetitive DNA sequence. FEMS Microbiol Lett 1996;137:207-11.
7. Spano F, Putignani L, McLauchlin J, Casemore DP, Crisanti A. PCR-RFLP analysis of the *Cryptosporidium* oocyst wall protein (COWP) gene discriminates between *C. wrairi* and *C. parvum*, and between *C. parvum* isolates of human and animal origin. FEMS Microbiol Lett 1997;150:209-17.
8. Arrowood MJ, Sterling CR. Isolation of *Cryptosporidium* oocysts and sporozoites using discontinuous sucrose and isopycnic Percoll gradients. J Parasitol 1987;73:314-9.
9. Arrowood MJ, Donaldson K. Improved purification methods for calf-derived *Cryptosporidium parvum* oocysts using discontinuous sucrose and cesium chloride gradients. J Eukaryot Microbiol 1996;43:S89.
10. Kim K, Gooze L, Petersen C, Gut J, Nelson RG. Isolation, sequence and molecular karyotype analysis of the actin gene of *Cryptosporidium parvum*. Mol Biochem Parasitol 1992;50:105-14.
11. Wisconsin Package [Computer program]. Version 9.0. Madison (WI): Genetics Computer Group; 1996.
12. Sanger F, Miklen S, Coulson AR. DNA sequencing with chain-terminating inhibitors. Proc Natl Acad Sci U S A 1977;74:5463-7.
13. Mead JR, Arrowood MJ, Sidwell RW, Healey MC. Chronic *Cryptosporidium parvum* infections in congenitally immunodeficient SCID and nude mice. J Infect Dis 1991;163:1297-304.
14. Arrowood MJ, Hurd MR, Mead JR. A new method for evaluating experimental cryptosporidial parasite loads using immunofluorescent flow cytometry. J Parasitol 1995;81:404-9.
15. Ong CSL, Pearce M, Eisler D, Goh SH, King AS, Bowie WR, and Isaac-Renton JL. An outbreak of cryptosporidiosis in southeastern British Columbia, Canada. In: Proceedings of the Symposium on Waterborne *Cryptosporidium* American Water Works Association. In press 1997.
16. Millard PS, Gensheimer KF, Addiss DG, Sosin DM, Beckett GA, Houck-Jankoski A, Hudson A. An outbreak of crytposporidiosis from fresh-pressed apple cider. JAMA 1994;272:1592-6.
17. Beach MJ, McNeil M, Arrowood M, Kreckman L, Craig A, Donaldson K, et al. *Cryptosporidium* in a water park: largest U.S. recreational waterborne outbreak [abstract]. 45th Annual Epidemic Intelligence Service Conference, Atlanta. 1996.
18. Centers for Disease Control and Prevention. Outbreak of cryptosporidiosis at a day camp—Florida, July-August 1995. MMWR Morb Mortal Wkly Rep 1996;45:442-4.
19. Addiss DG, Mac Kenzie WR, Hoxie NJ, Gradus MS, Blair KA, Proctor JE, et al. Epidemiologic features and implications of the Milwaukee cryptosporidiosis outbreak. In: Betts WB, Casemore D, Fricker C, Smith H, Watkins J, editors. Protozoan parasites and water. Cambridge, United Kingdom: Royal Society of Chemistry; 1995. p. 19-25.
20. Awad-El-Kariem FM, Robinson HA, Dyson DA, Evans D, Wright S, Fox MT, McDonald V. Differentiation between human and animal strains of *Cryptosporidium parvum* using isoenzyme typing. Parasitology 1995;110:129-32.
21. Vasquez JR, Gooze L, Kim K, Gut J, Petersen C, Nelson RG. Potential antifolate resistance determinants and genotypic variation in the bifunctional dihydrofolate reductase-thymidylate synthase gene from human and bovine isolates of *Cryptosporidium parvum*. Mol Biochem Parasitol 1996;79:153-65.
22. Pozio E, Gomez Morales MA, Barbieri FM, La Rosa G. *Cryptosporidium*: different behaviour in calves of isolates of human origin. Trans R Soc Trop Med Hyg 1992;86:636-8.
23. Hojlyng N, Molbak K, Jepsen S. Cryptosporidiosis in human beings is not primarily a zoonosis. J Infect 1985;11:270-2.
24. Robson KJH, Hall JRS, Davies LC, Crisanti A, Hill AVS, Wellems TE. Polymorphism of the TRAP gene of *Plasmodium falciparum*. Proc R Soc Lond B Biol Sci 1990;242:205-16.
25. Muller HM, Scarselli E, Crisanti A. Thrombospondin related anonymous protein (TRAP) of *Plasmodium falciparum* in parasite-host cell interactions. Parassitologia 1993;35 (suppl):69-72.
26. Templeton TJ, Kaslow DC. Cloning and cross-species comparison of the thrombospondin-related anonymous protein (TRAP) gene from *Plasmodium knowlesi*, *Plasmodium vivax* and *Plasmodium gallinaceum*. Mol Biochem Parasitol 1997;84:13-24.

## References

[PDF_00569] Arrowood M. J., Hurd M. R., Mead J. R. (1995). A new method for evaluating experimental cryptosporidial parasite loads using immunofluorescent flow cytometry.. J Parasitol.

[PDF_00549] Arrowood M. J., Sterling C. R. (1987). Isolation of Cryptosporidium oocysts and sporozoites using discontinuous sucrose and isopycnic Percoll gradients.. J Parasitol.

[PDF_00599] Awad-el-Kariem F. M., Robinson H. A., Dyson D. A., Evans D., Wright S., Fox M. T., McDonald V. (1995). Differentiation between human and animal strains of Cryptosporidium parvum using isoenzyme typing.. Parasitology.

[PDF_00537] Bonnin A., Fourmaux M. N., Dubremetz J. F., Nelson R. G., Gobet P., Harly G., Buisson M., Puygauthier-Toubas D., Gabriel-Pospisil G., Naciri M. (1996). Genotyping human and bovine isolates of Cryptosporidium parvum by polymerase chain reaction-restriction fragment length polymorphism analysis of a repetitive DNA sequence.. FEMS Microbiol Lett.

[PDF_00588] Centers for Disease Control and Prevention (CDC) (1996). Outbreak of cryptosporidiosis at a day camp--Florida, July-August 1995.. MMWR Morb Mortal Wkly Rep.

[PDF_00516] Colford J. M., Tager I. B., Hirozawa A. M., Lemp G. F., Aragon T., Petersen C. (1996). Cryptosporidiosis among patients infected with human immunodeficiency virus. Factors related to symptomatic infection and survival.. Am J Epidemiol.

[PDF_00514] Guerrant R. L. (1997). Cryptosporidiosis: an emerging, highly infectious threat.. Emerg Infect Dis.

[PDF_00614] Højlyng N., Mølbak K., Jepsen S. (1985). Cryptosporidiosis in human beings is not primarily a zoonosis.. J Infect.

[PDF_00556] Kim K., Goozé L., Petersen C., Gut J., Nelson R. G. (1992). Isolation, sequence and molecular karyotype analysis of the actin gene of Cryptosporidium parvum.. Mol Biochem Parasitol.

[PDF_00520] Mac Kenzie W. R., Hoxie N. J., Proctor M. E., Gradus M. S., Blair K. A., Peterson D. E., Kazmierczak J. J., Addiss D. G., Fox K. R., Rose J. B. (1994). A massive outbreak in Milwaukee of cryptosporidium infection transmitted through the public water supply.. N Engl J Med.

[PDF_00565] Mead J. R., Arrowood M. J., Sidwell R. W., Healey M. C. (1991). Chronic Cryptosporidium parvum infections in congenitally immunodeficient SCID and nude mice.. J Infect Dis.

[PDF_00579] Millard P. S., Gensheimer K. F., Addiss D. G., Sosin D. M., Beckett G. A., Houck-Jankoski A., Hudson A. (1994). An outbreak of cryptosporidiosis from fresh-pressed apple cider.. JAMA.

[PDF_00525] Morgan U. M., Constantine C. C., O'Donoghue P., Meloni B. P., O'Brien P. A., Thompson R. C. (1995). Molecular characterization of Cryptosporidium isolates from humans and other animals using random amplified polymorphic DNA analysis.. Am J Trop Med Hyg.

[PDF_00621] Müller H. M., Scarselli E., Crisanti A. (1993). Thrombospondin related anonymous protein (TRAP) of Plasmodium falciparum in parasite-host cell interactions.. Parassitologia.

[PDF_00610] Pozio E., Gomez Morales M. A., Barbieri F. M., La Rosa G. (1992). Cryptosporidium: different behaviour in calves of isolates of human origin.. Trans R Soc Trop Med Hyg.

[PDF_00617] Robson K. J., Hall J. R., Davies L. C., Crisanti A., Hill A. V., Wellems T. E. (1990). Polymorphism of the TRAP gene of Plasmodium falciparum.. Proc Biol Sci.

[PDF_00562] Sanger F., Nicklen S., Coulson A. R. (1977). DNA sequencing with chain-terminating inhibitors.. Proc Natl Acad Sci U S A.

[PDF_00543] Spano F., Putignani L., McLauchlin J., Casemore D. P., Crisanti A. (1997). PCR-RFLP analysis of the Cryptosporidium oocyst wall protein (COWP) gene discriminates between C. wrairi and C. parvum, and between C. parvum isolates of human and animal origin.. FEMS Microbiol Lett.

[PDF_00625] Templeton T. J., Kaslow D. C. (1997). Cloning and cross-species comparison of the thrombospondin-related anonymous protein (TRAP) gene from Plasmodium knowlesi, Plasmodium vivax and Plasmodium gallinaceum.. Mol Biochem Parasitol.

[PDF_00604] Vásquez J. R., Goozé L., Kim K., Gut J., Petersen C., Nelson R. G. (1996). Potential antifolate resistance determinants and genotypic variation in the bifunctional dihydrofolate reductase-thymidylate synthase gene from human and bovine isolates of Cryptosporidium parvum.. Mol Biochem Parasitol.

